# Measuring phenotype-phenotype similarity through the interactome

**DOI:** 10.1186/s12859-018-2102-9

**Published:** 2018-04-11

**Authors:** Jiajie Peng, Weiwei Hui, Xuequn Shang

**Affiliations:** School of Computer Science, Northwestern Polytechnical University, Xi’an, China

**Keywords:** Phenotype relationships, Interactome, Human phenotype ontology

## Abstract

**Background:**

Recently, measuring phenotype similarity began to play an important role in disease diagnosis. Researchers have begun to pay attention to develop phenotype similarity measurement. However, existing methods ignore the interactions between phenotype-associated proteins, which may lead to inaccurate phenotype similarity.

**Results:**

We proposed a network-based method *PhenoNet* to calculate the similarity between phenotypes. We localized phenotypes in the network and calculated the similarity between phenotype-associated modules by modeling both the inter- and intra-similarity.

**Conclusions:**

*PhenoNet* was evaluated on two independent evaluation datasets: gene ontology and gene expression data. The result shows that *PhenoNet* performs better than the state-of-art methods on all evaluation tests.

## Background

Recently, advances in next generation sequencing (NGS) have significantly improved the Mendelian disease diagnosis [1–4]. However, disease diagnosis only using sequence-based approach is still challenging, since lots of diseases have complex phenotypes and high genetic heterogeneity. It is difficult to reveal the relationship between genetic features and complex patient phenotypic features [5].

In medical contexts, “phenotype” often refers to the deviation from normal morphology, physiology, or behavior [6]. Phenotype carries the biologically meaningful information [7, 8]. Disease is usually the result of congenital or acquired mutations [9, 10], which causes the phenotypes of diseases [11]. Therefore, phenotype plays an important role in disease diagnosis process. Clinical practice and medical research based on phenotype analysis have drawn great attention in recent years [12–14]. Measuring phenotype similarity became one of the key components in disease diagnosis and understanding the disease mechanism [15].

In the past few years, a lot of computational methods have been proposed to calculate phenotype similarity [7, 11, 16–24]. These approaches can be loosely grouped into two categories: text mining-based method and ontology-based method. Text mining-based methods measure the phenotype similarity by comparing texts describing phenotypes. These methods extract features from the descriptions and construct machine learning models [25, 26] based on these features [7, 8]. Driel et al. classified more than 5000 phenotypes based on texts from the OMIM database [7]. They found the similarity between phenotypes are positively correlated with protein sequence similarity and protein-protein interaction. However, text data always results to ambiguous meanings. For example, different words may represent the same meaning.

To avoid ambiguous representation, a unified vocabulary system was developed to describe phenotype, named Human Phenotype Ontology (HPO). HPO is widely used to describe human phenotypic abnormalities using a structured, controlled and unified vocabularies, which are constructed as a directed acyclic graph (DAG). The ontology-based method measures the phenotype similarity based on the HPO. HPO-based semantic similarity has been used to quantify the phenotypic similarity between patient symptoms and known phenotypes related to a gene [27, 28]. This type of method is based on hierarchical structure of HPO and the annotations of phenotypes [11, 29–33]. Phenomizer and Masino et al. calculated the similarity between phenotypes based on the information content (IC) of their lowest common-ancestor in HPO [27, 28]. Given a term *t*, its IC is calculated as: $IC(t) = -log \frac {|D_{t}|}{D}$, where *D*_*t*_ and *D* represent annotation set of *t* and all annotations involved in HPO respectively. Performance evaluation shows that this method performs better than the term matching-based approach that ignores the semantic relation between terms. However, this method may be hindered by the noises in the patient phenotype data [28]. A PageRank-based method, named *PhenoSim*, was proposed to model the noises in the patient phenotype data set before applying the ontology-based method [23]. The evaluation test shows that *PhenoSim* performs better than the IC-based method. However, the ontology-based method relies on the hierarchical structure of HPO, which makes it hard to distinguish the similarities between terms that have the same lowest common ancestor. For example, let *A*, *B* and *C* be terms with the same common ancestor. The ontology-based method can not measure whether similarity of *A* and *B* are higher than *B* and *C*. Furthermore, aforementioned existing methods ignores the interactions between the proteins annotated by the phenotypes, which is critical to understand the molecular basis of phenotypes.

Recently, network-based method has been proposed to measure the similarity between diseases [20, 34–36]. The similarity between two diseases is measured by exploring the relationships between disease-associated proteins within the interactome. Specifically, given disease *d*_*a*_, *d*_*b*_ and their associated gene set *G*_*a*_ and *G*_*b*_, the similarity between *d*_*a*_ and *d*_*b*_ is calculated as follows.

1 $$ \ {s}_{ab} = h_{ab} - \frac{h_{aa} + h_{bb}}{2}  $$

where *h*_*ab*_ is the average of shortest distances of protein pairs across *G*_*a*_ and *G*_*b*_. *h*_*aa*_ (*h*_*bb*_) is the average of shortest distances in *d*_*a*_ (*d*_*b*_). The evaluation test shows that the network-based similarities have high correlation with gene co-expression patterns, symptom similarities and gene ontology-based similarities [20].

Unfortunately, to our knowledge, the network-based model has not been used to measure the similarity between phenotypes. To overcome the disadvantages in aforementioned phenotype similarity measurements, we present a network-based method, called *PhenoNet*, to calculate the phenotype similarity by comparing the phenotype-associated modules in the protein-protein interaction network. We localize the phenotypes in the network and propose a network-based method considering both intra- and inter-similarity of the phenotype-associated modules, which can effectively use the rich information in the network to measure the phenotype similarity. Comparing with the existing approaches, the contributions of our work are listed as follows:

- Our work indicates that phenotypes can be represented by the network modules.
- To the best of our knowledge, *PhenoNet* is the first phenotype similarity measurement based on the interactome.
- We proposed a novel method to calculate similarity between phenotypes considering both the inter- and intra-similarity of the phenotype-associated module in the network.

## Methods

**Fig. 1:**
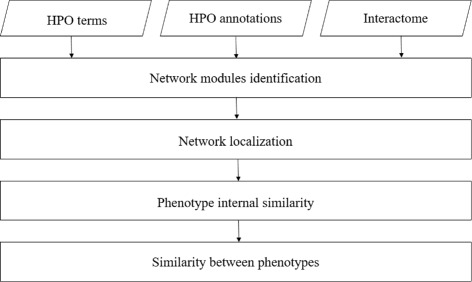
The workflow of *PhenoNet*

We propose a new network-based method called *PhenoNet* to measure the relationships between phenotypes based on the biological network. Our model includes four parts. Firstly, for each given phenotype *p*, we identify the corresponding module in the given biological network, labeled as *n*_*p*_. Secondly, statistical method is used to test whether phenotypes could be represented by network modules. Thirdly, for each network module *n*_*p*_, we first calculate the internal similarity by modeling the relationships among nodes in *n*_*p*_. Finally, given two phenotypes *p*_1_ and *p*_2_, their relationship is measured based on corresponding network module $n_{p_{1}}$ and $n_{p_{2}}$. The diagram of the whole process is shown in Fig. 1.

### Network modules identification

In order to measure the relationship between two phenotypes based on a biological network *N*, the first step is to identify network modules in *N* to represent phenotypes. In this step, we will generate a set of network modules corresponding to a set of phenotypes. In general, network module means a group of nodes that associate with a specific function, disease or phenotype *etc.* Given a biological network *N*, our goal is to identify a network module corresponding to a phenotype *p*.

HPO is constructed as a directed acyclic graph (DAG), in which each term represents a phenotype. Each phenotype term *p* is related to a set of proteins *G*_*p*_, which can be used to identify the phenotype module in a given biological network. Specifically, given a biological network *N*(*V,E*), a phenotype *p* and a set of proteins *G*_*p*_ related to *p*, the network module of *p* in *N* is a subnetwork of *N*, labeled as $\phantom {\dot {i}\!}N_{p} (V^{\prime },E^{\prime })$. *V*^′^ is the intersection set of *V* and *G*_*p*_. *E*^′^ is a subset of *E*, which connects the nodes in *E*^′^.

### Network localization

Given a network module, we test whether proteins in a network module can agglomerate in specific interactome neighborhoods. If a network module of a phenotype is a highly interconnected group of proteins, the phenotype could be represented by a network module. Then, we could measure the relations between phenotypes by comparing network modules that represent phenotypes.

**Fig. 2:**
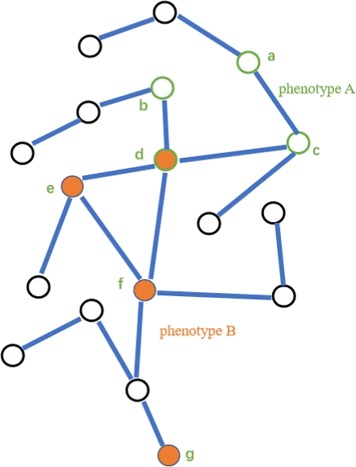
An illustration example of protein-protein interaction network. In this network, network module of phenotype *A* consists of the nodes with green circle. Network module of phenotype *B* consists of the nodes filled with orange. Specifically, phenotype *B* is split into two components

We used two metrics to quantify the localization of the identified network module. One is the module size. Module size is defined as the number of proteins contained in the largest connected subnetwork of a network module. The other is the shortest distance. For each protein, the shortest distance is defined as the distance to the closest protein in the same network module. Given a network module *N*_*A*_ of phenotype *A*, the shortest distance is defined as follows.

2 $$ d_{A} = \frac{\sum_{v \in N_{A}}d_{v}}{|N_{A}|}  $$

where *d*_*v*_ is the shortest distance of a protein *v* in the network module *N*_*A*_. In the illustrative example in Fig. 2, the module sizes of network modules corresponding to phenotype *A* and *B* are 4 and 3 respectively. The shortest distances of network modules corresponding to phenotype *A* and *B* are 4 and 5 respectively.

To test whether the proteins in a network module agglomerate together, we compare the module size and shortest distance of a network module with a random control. Given a phenotype *A* and its corresponding network module *N*_*A*_, we randomize the phenotype associations of proteins and generate a random network module *NA*′ on the given PPI network. *NA*′ includes the same number of proteins as *N*_*A*_. Given a phenotype, we repeat this procedure 100,000 times to obtain 100,000 network modules corresponding to *A*. Based on the randomly generated network modules, we can calculate the statistical significance of the real data. For both module size and shortest distance, we estimate the mean values and standard deviations. Given a network module *N*_*A*_, the *z*−*score* of module size is calculated as follows.

3 $$ {z^{size}_{A}} = \frac{{s_{A}} - {\mu_{1}}}{{\sigma_{1}}}  $$

where *s*_*A*_ is the module size of *N*_*A*_. *μ*_1_ and *σ*_1_ represent the mean value and standard deviation of module size based on the randomly generated network modules.

Similarly, the *z*−*score* of shortest distance is calculated as follows.

4 $$ {z^{dis}_{A}} = \frac{{d_{N_{A}}} - {\mu_{2}}}{{\sigma_{2}}}  $$

where $d_{N_{A}}$ is the shortest path of *N*_*A*_. *μ*_2_ and *σ*_2_ represent the mean value and standard deviation of shortest path based on the randomly generated network modules.

We could also obtain the corresponding *p*-value for each *z*−*score* after calculating the *z*−*score* for every network module identified in the network. Therefore, we can test whether proteins in a identified network module are highly interconnected via the statistical significance of module size and shortest distance.

### Phenotype internal similarity calculation

The similarity between two phenotypes could be measured by measuring the distance between their corresponding network modules in a network. To calculate the similarity between two network modules, we consider both the internal similarity in each network module and similarity between network modules. The idea is inspired by the traditional clustering evaluation method that both inter- and intra- similarity should be considered. Given two network modules *N*_*A*_ and *N*_*B*_, their similarity in the network is proportional to the intra-similarity of *N*_*A*_ (*N*_*B*_) and the inter-similarity between *N*_*A*_ and *N*_*B*_.

The intra-similarity of phenotype *N*_*A*_ is defined as the average of pairwise similarities between proteins contained in *N*_*A*_. The similarity between two proteins *i* and *j* is defined as follows.

5 $$ Sim(i,j) = \frac{1}{d_{s}(i,j)}  $$

where *d*_*s*_(*i,j*) is the length of the shortest path between *i* and *j*. Mathematically, the intra-similarity of network module *N*_*A*_ is defined as follows.

6 $$ {{{Sim}_{intra}(N_{A})}} = \frac{\sum_{i \in N_{A}}\sum_{j \in N_{A}, j\neq i}{Sim} {(i,j)}}{{|N_{A}|} \times (|N_{A}|-1)}  $$

The intra-similarity of a network module can be calculated based on Eq. 6. Intra-similarity can reflect whether a network module agglomerate together in the network.

### Similarity between phenotypes calculation

The network-based similarity of phenotype *A* and *B* can be quantified by comparing the similarity between network module *N*_*A*_ and *N*_*B*_ corresponding to *A* and *B* respectively.

As we described previously, the similarity between two network modules is determined by both intra- and inter- similarity. The inter-similarity between phenotype *N*_*A*_ and *N*_*B*_ is calculated based on pairwise similarities between proteins in different network modules. Let *i* be a protein involved in *N*_*A*_, the similarity between *i* and *N*_*B*_ is computed with the following equation:

7 $$ \mathit{{S_{i\rightarrow N_{B}}}} = \left\{ \begin{array}{lr} \frac{\sum_{j \in N_{B}} Sim {(i,j)}}{|N_{B}|}, & i \neq j \\ 1, & i=j \end{array} \right.  $$

where *Sim*(*i*,*j*) is the similarity between protein *i* and *j* (see Eq. 5), |*N*_*B*_| represents the number of proteins involved in the network module *N*_*B*_. Particularly, if *i* and *j* are identical, the similarity value is set as 1. Then, we can calculate the inter-similarity between two network modules *N*_*A*_ and *N*_*B*_ with following equation:

8 $$ {\begin{aligned} {{Sim}_{inter}(N_{A},N_{B})} = \frac{1}{2}\left(\frac{\sum_{i \in N_{A}}S_{i\rightarrow N_{B}}}{|N_{A}|} + \frac{ \sum_{i \in N_{B}}S_{i\rightarrow |N_{A}|}}{|N_{B}|}\right) \end{aligned}}  $$

where |*N*_*A*_| and |*N*_*B*_| represent the number of proteins involved in the network module *N*_*A*_ and *N*_*B*_ respectively. Equation 8 includes two parts: the average of similarities between each protein *i*∈*N*_*A*_ and *N*_*B*_; the average of similarities between each protein *i*∈*N*_*B*_ and *N*_*A*_. Note that the aforementioned two parts are asymmetric. To avoid the asymmetry result, the inter-similarity of two network modules are calculated as Eq. 8.

By considering both inter- and intra- similarity, the similarity between two network module *N*_*A*_ and *N*_*B*_ corresponding to phenotype *A* and *B* is calculated as follows.

9 $$ {\begin{aligned} {Sim(A,B)} \,=\, {{Sim}_{inter}(N_{A},N_{B})} \,-\, \frac{{{Sim}_{intra}(N_{A}) \,+\, {Sim}_{intra}(N_{B})}}{2} \end{aligned}}  $$

where *A* and *B* are two phenotypes, *N*_*A*_ and *N*_*B*_ are two network modules in the PPI network corresponding to *A* and *B* respectively.

## Results and discussion

### PhenoNet implementation and data preparation

*PhenoNet* was implemented with Python 2.7 and *networkx* library. HPO data was downloaded from the HPO website in Apr. 2016 (http://human-phenotype-ontology.github.io/downloads.html). HPO data contains 11786 human phenotype terms. There exist relationships (is_a) between HPO terms. We used this relationship to generate HPO term tree firstly. Then we up-propagated the HPO terms with the hierarchy of the full HPO tree. From the phenotype terms we selected the HPO terms with at least 25 genes based on the percolation theory, for the reason that our protein-protein interaction network is incomplete. Since our goal is to compute the similarity between different phenotypes, we deleted all the parent terms from the term sets obtained in the former step in order to eliminate the influence from parent-son relationships. Finally we used 1061 HPO terms in our experiment.

GO data was downloaded from GO website in Nov. 2016 (www.geneontology.org/). We only used the annotations with high confidence. Specifically, the selected annotations are associated with the following evidence codes: EXP, IDA, IMP, IGI, IEP, ISS, ISA, ISM and ISO. And we ignored the annotations with evidence code IPI to avoid the logical cycle of the performance evaluation, since the protein-protein interaction network that used to calculate the phenotype similarity is constructed by the physical protein-protein interactions. We also removed the annotations with a non-empty “qualifier” column [37]. The annotations of a GO term includes its direct annotations and annotations of its descendants.

The gene expression data was from (http://www.ncbi.nlm.nih.gov/geo, downloaded Jan. 2017) [38]. We excluded nine unhealthy tissue data of the 79 tissues in the dataset [20]. The highest expression value is selected when an mRNA has multiple transcripts. After mapping mRNA to gene, we totally obtained 2849 genes with expression value.

The interactome generated by Menche et al. was used in the following tests [20], which integrated several databases with seven types of physical interactions: regulatory interactions [39], binary interactions [40–44], literature curated interactions [45–47], metabolic enzyme-coupled interactions [48], protein complexes [49], kinase network (kinase-substrate pairs) [50] and signaling interactions [51]. The final PPI interaction network includes 141,296 interactions between 13,460 proteins. Note that the interactions extracted from gene expression data or evolutionary considerations are not included.

### Network localization

**Fig. 3:**
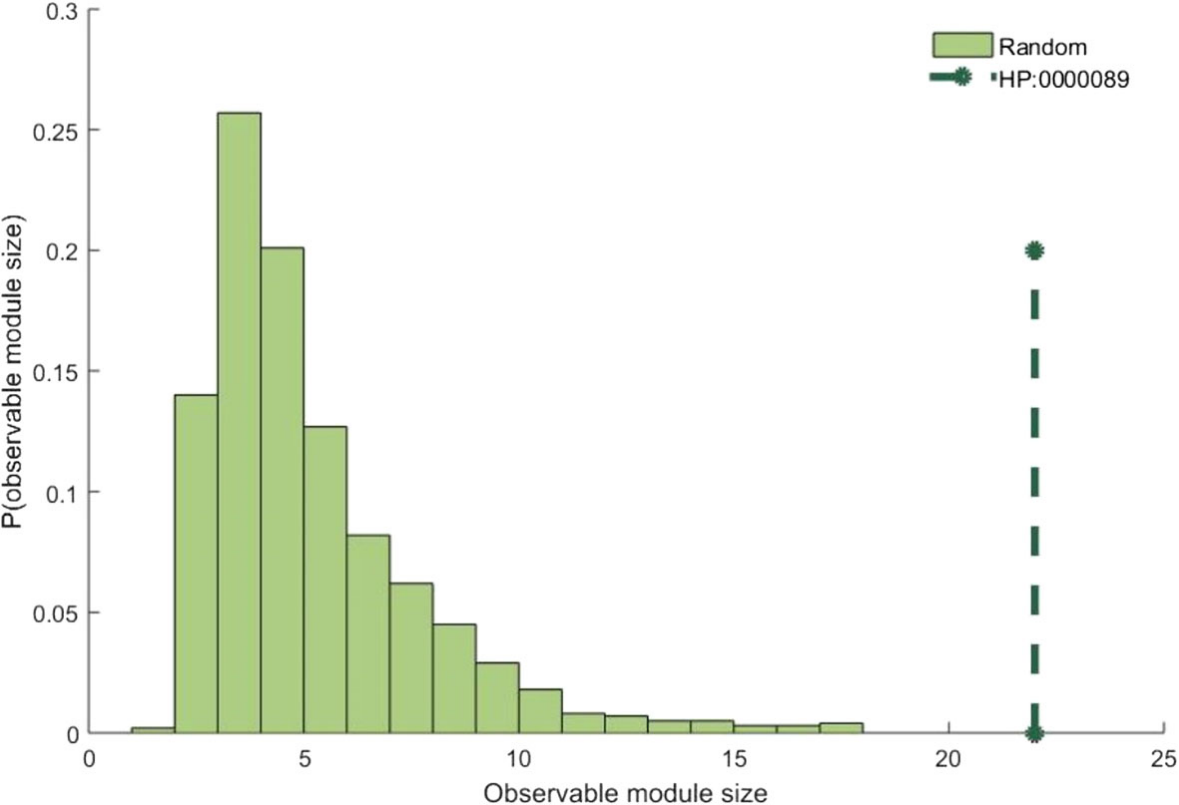
Module size distribution of the random models based on phenotype “HP:0000089”. The module size of random models is less than the phenotype

**Fig. 4:**
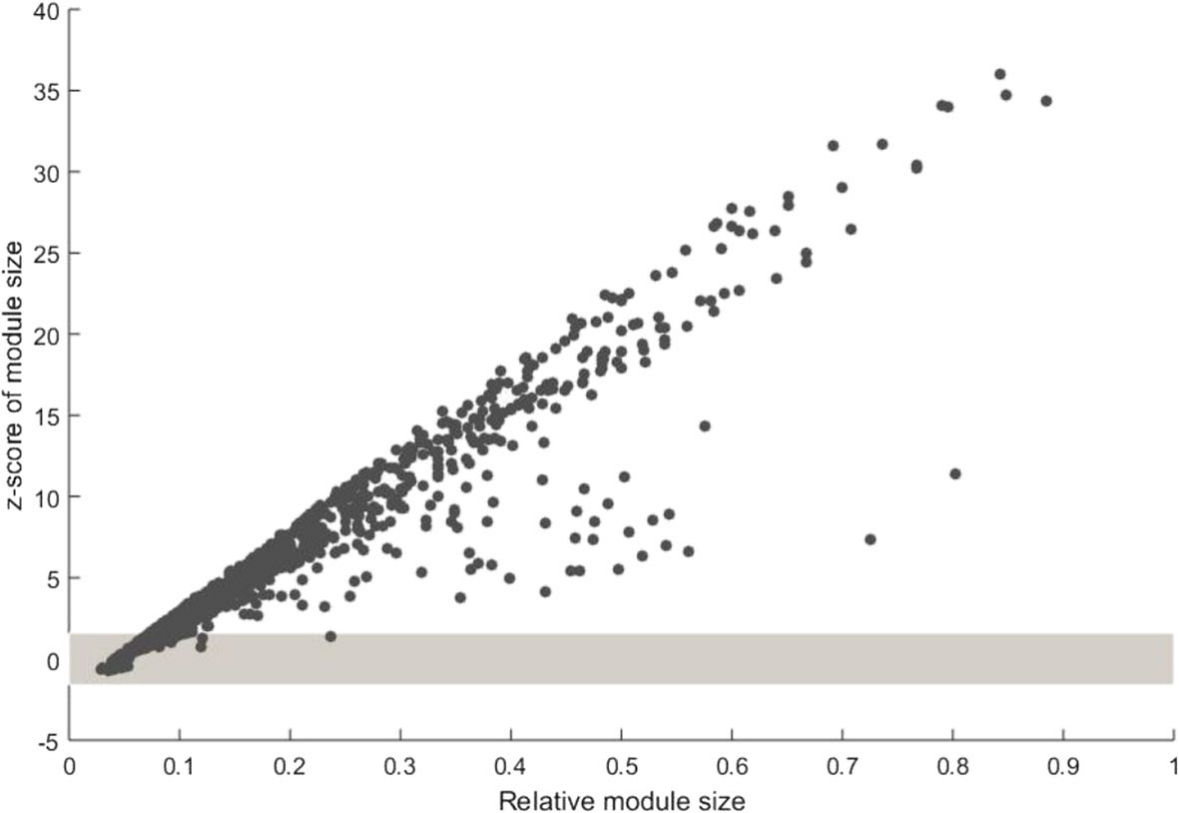
*z*−*score* of 1061 HPO terms based on their module size. Eight hundred seventy two phenotypes are significant. Points in the gray area represent the phenotypes which are not significant

We used phenotype module size and the shortest distance to test whether these phenotype proteins tend to agglomerate in the protein-protein interaction network. Taking phenotype “Renal hypoplasia (HP: 0000089)” as an example, the average module size of the randomly generated models is significantly less than the real module size (the number is 22) of term HP: 0000089 (Fig. 3, *p*-value ≤10^−5^, the threshold of *z*−*score* is *z*−*score*≥ 1.6, *p*-value ≤ 0.05). Overall, 872 of 1061 phenotypes are statistically significant based on module size. The *z*−*score* based on the module sizes of these phenotypes are shown in Fig. 4. It shows that the module sizes of most phenotypes are significant, indicating that proteins involved in the same phenotype tend to be connected.

**Fig. 5:**
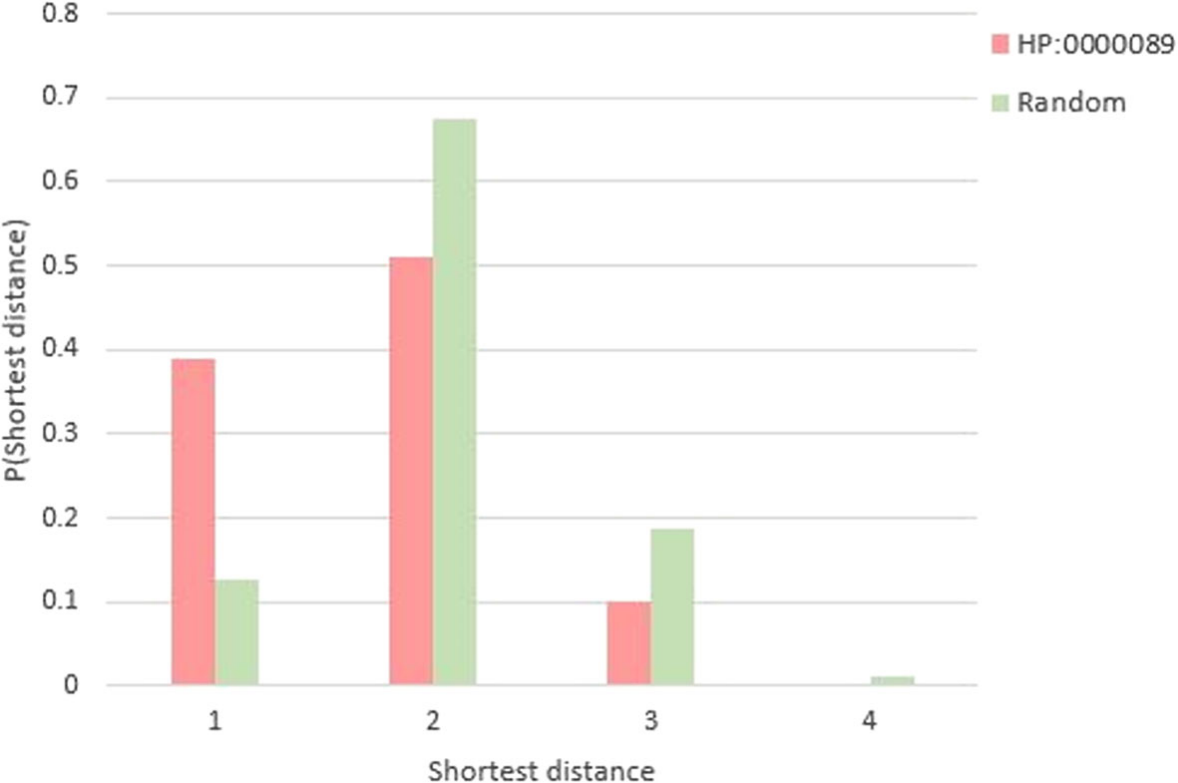
Shortest distance distribution of the random set based on HP:0000089. The shortest distance of phenotype “HP:0000089” is smaller than the randomly set. The x-axis is the shortest distance. The y-axis is the probability of each shortest distance. Let *s* be the size of HPO, *n*_*i*_ be the number of shortest distance *i* (*i* can be 1,2,3,4), then the probability is calculated by $\frac { n_{i}}{100000 * s} $

**Fig. 6:**
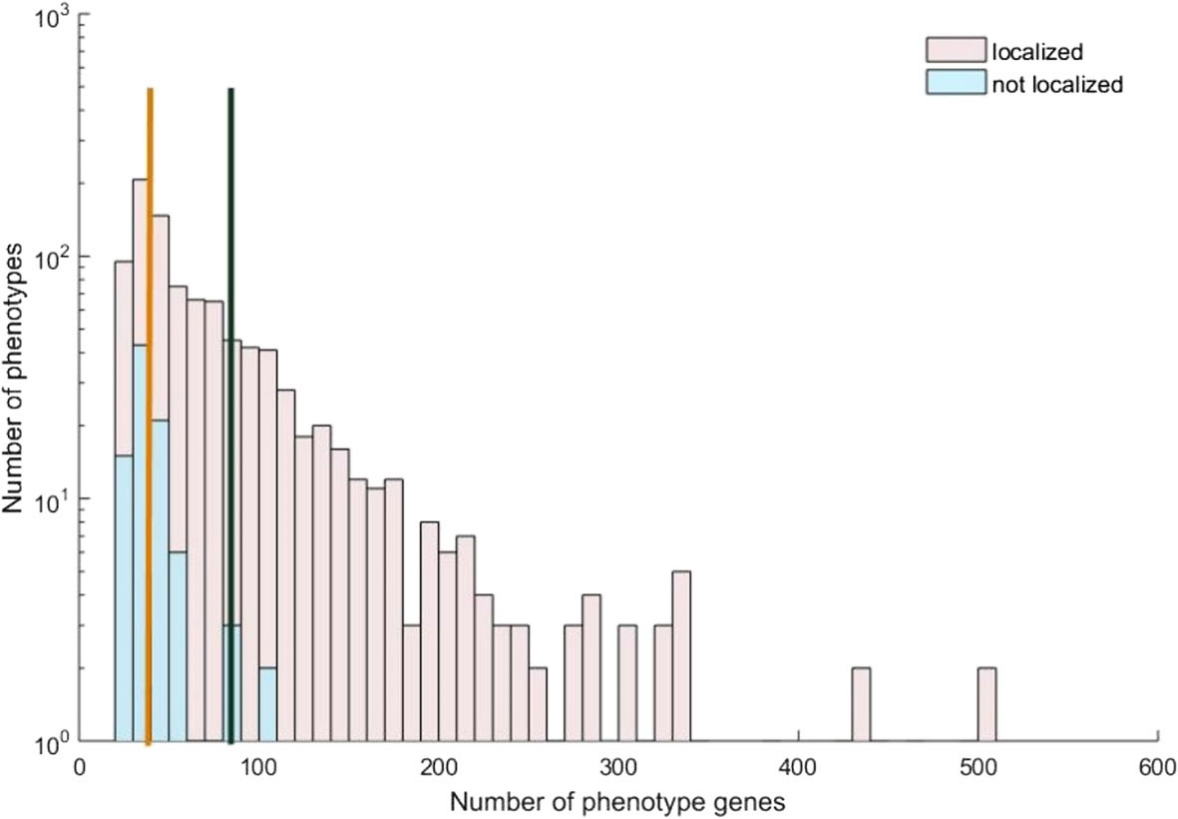
Distribution for *z*−*score* of 1061 HPO terms on shortest distance. The pink and blue bars represent the distribution of localized and not-localized respectively. The average numbers of proteins associated with localized and not-localized phenotypes are 82 and 40 respectively

For the shortest distance, we also take phenotype “HP: 0000089” as an example. Figure 5 shows that the shortest distance of the randomly generated modules is significantly larger than the real phenotype “HP: 0000089” (*z*−*score*= -5.51, *p*-value ≤10^−5^). The results indicate that proteins associated with one phenotype are usually a group of interconnected nodes in the network instead of dispersing across the whole network. Overall, 969 of 1061 phenotypes have significantly shorter distances compared to the random distribution (Fig. 6). The black and yellow line in the figure show the average number of phenotype proteins of the well-localized phenotypes and not-localized phenotypes (82 and 40 respectively).

In summary, the proteins involved in a phenotype have shown a statistically significant tendency to agglomerate together in the network based on both module size and shortest distance.

### Performance evaluation on gene ontology dataset

To evaluate the performance of *PhenoNet*, we employed GO-based similarity as the independent evidence to compare the performance of *PhenoNet* with two existing algorithms, i.e. ontology-based method [29] and Menche’s method [20]. Ontology-based method calculated the phenotype similarity based on the IC of the lowest common ancestor of the phenotypes in HPO. Menche’s method [20] is a network-based method which was used to measure the disease similarity.

The idea is that the network-based similarity between phenotypes should be correlated with the GO-based similarity. Given two phenotypes *p*_1_ and *p*_2_, let *G*_1_ and *G*_2_ be the protein sets associated with *p*_1_ and *p*_2_ respectively. By adopting the method used in [20], the similarity between *p*_1_ and *p*_2_ is measured by the average of all protein pairs between *G*_1_ and *G*_2_ (Equation 10).

10 $$ {{Sim}_{phe}(p1,p2)} = \frac{1}{{|G_{1}|\times|G_{2}|}} \sum_{g_{i} \in G_{1}, g_{j} \in G_{2}} {{Sim}_{pro}(g_{i},g_{j})}  $$

*Sim*_*pro*_(*g*_*i*_,*g*_*j*_) is the functional similarity between *g*_*i*_ and *g*_*j*_ based on GO. It is defined based on the specificity of shared GO annotations as: ${Sim}_{pro}(g_{i},g_{j}) = \frac {2}{n_{t}}$. *n*_*t*_ is the total number of proteins annotated to the most specific GO term *t* shared by *g*_*i*_ and *g*_*j*_. Particularly, *Sim*_*pro*_(*g*_*i*_,*g*_*j*_)=1 if *g*_*i*_ and *g*_*j*_ are the only two proteins annotated by a specific GO term.

**Fig. 7:**
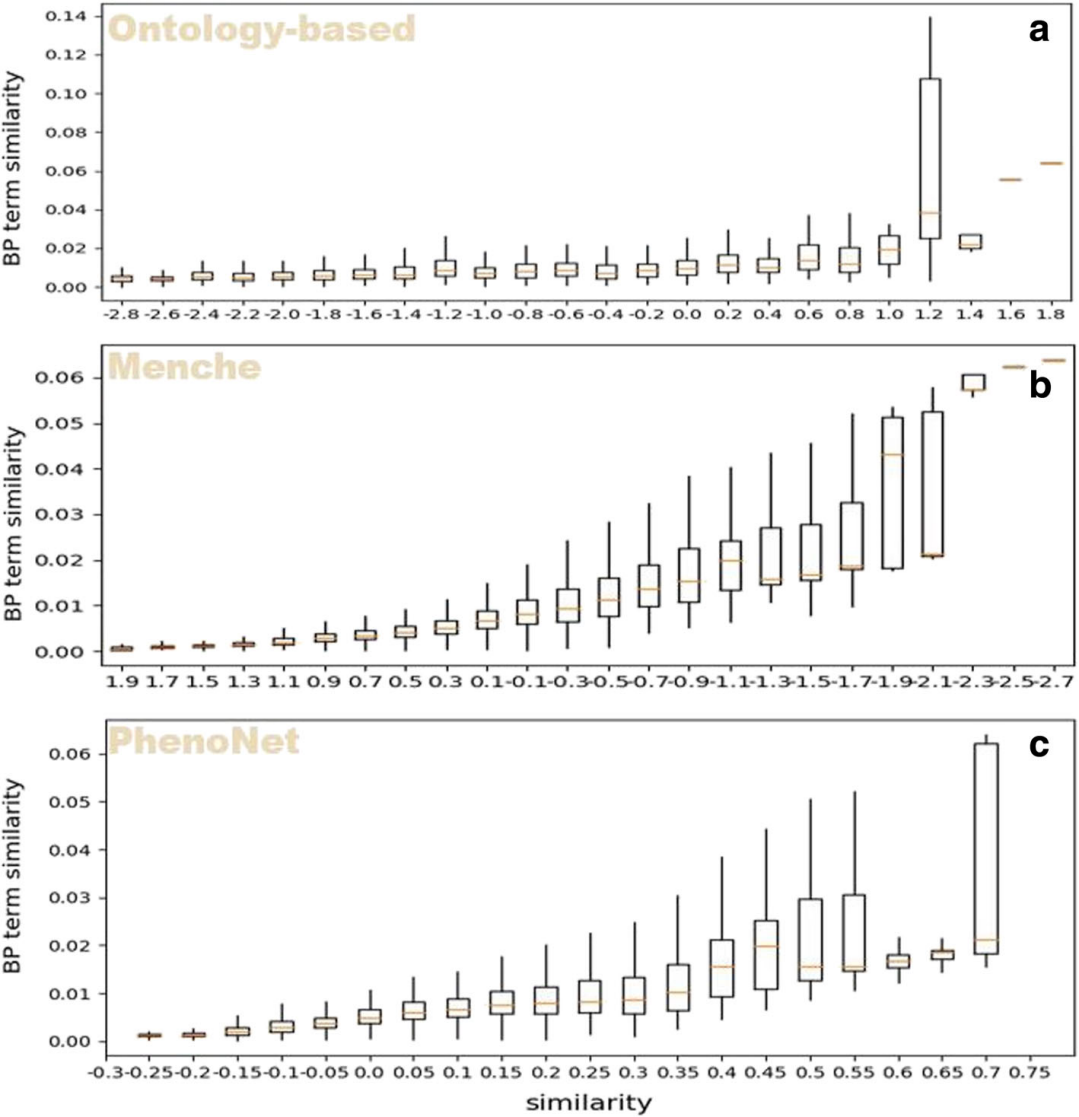
Phenotype similarity versus GO term similarity. The x-axis shows the similarity calculated by three methods: ontology-based (**a**), Menche’s method (**b**) and *PhenoNet* (**c**). The y-axis is GO term similarity based on biological process category

**Fig. 8:**
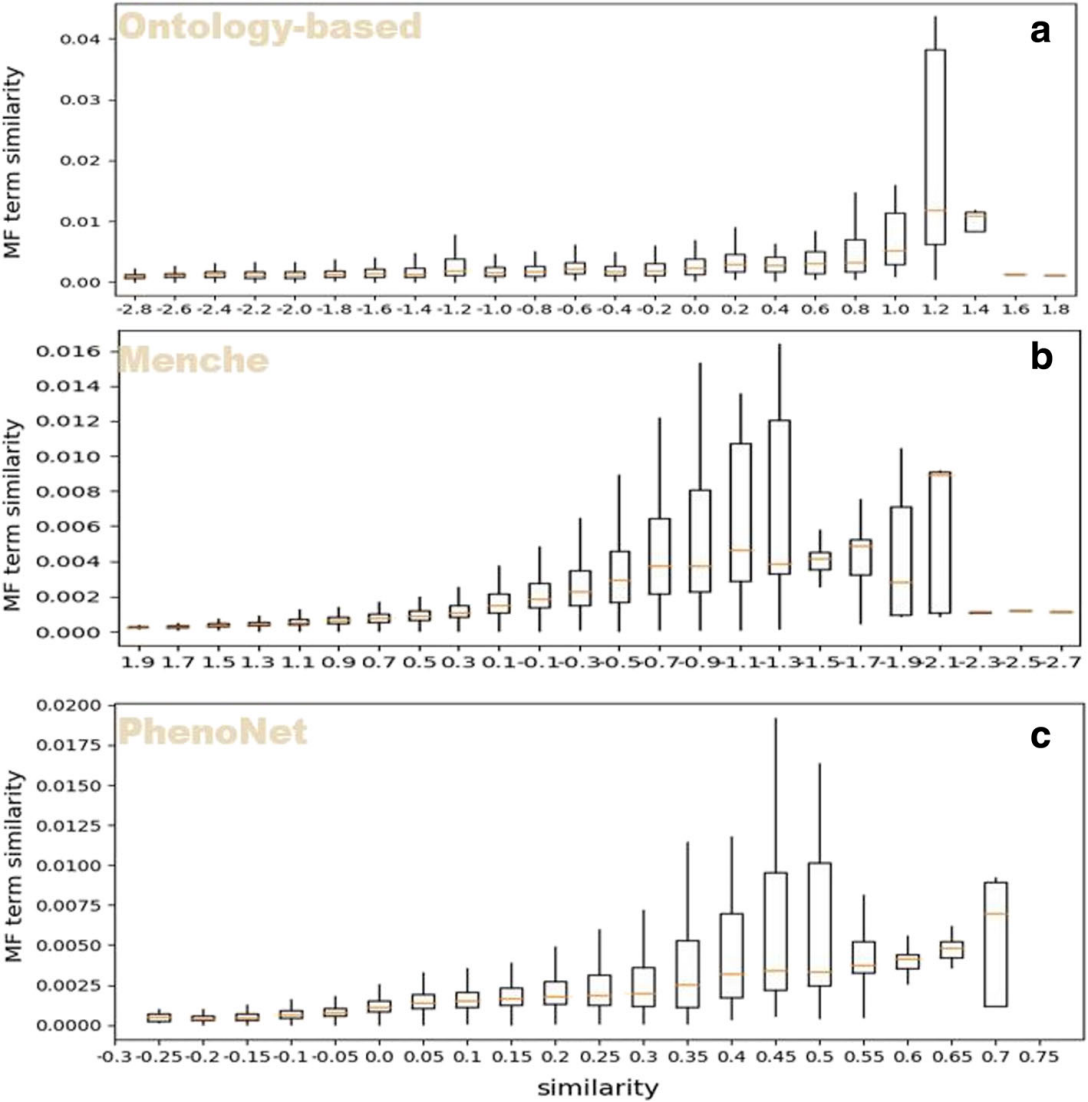
Phenotype similarity versus GO term similarity. The x-axis shows the similarity calculated by three methods: ontology-based (**a**), Menche’s method (**b**) and *PhenoNet* (**c**). The y-axis is GO term similarity based on molecular function category

With the similarity between phenotypes based on GO functional similarity, we can compare *PhenoNet* with other existing methods. Firstly, we tested the performance of *PhenoNet* with the functional similarity based on GO biological process category. In general, *PhenoNet* performs better than other methods (Table 1). Figure 7 shows that similarities based on all three methods are correlated with GO BP category-based similarities. Quantificationally, *Pearson* correlation coefficient (*PCC*) scores [52] of *PhenoNet* on median and mean value are both 0.96, which are higher than the second best method Menche’s (0.82 and 0.89). Furthermore, the *R*^2^ scores [53] of *PhenoNet* on the median and mean value are 0.93 and 0.92, which are 0.24 and 0.13 higher than the second best method Menche’s respectively. Similarly, we also compared the three methods based on the GO molecular function (MF) category. Figure 8 shows that only *PhenoNet* similarities increase steadily with the increase of the GO MF-based similarities. In general, the performance of *PhenoNet* is significantly higher than other methods. Specifically, *Pearson* correlation coefficient (*PCC*) scores of *PhenoNet* on the median and mean value are 0.94 and 0.95 respectively, which are 0.33 and 0.29 higher than the second best method Menche’s. In addition, the *R*^2^ scores of *PhenoNet* on median and mean value are more than two times of the second best method Menche’s, which are 0.37 and 0.44 respectively.

**Table 1:** The Pearson correlation coefficient and *R*^2^ score between three methods and GO-based similarity

GO term	Measure		Ontology-based	Menche	PhenoNet
BP	PCC	Median	0.58	0.82	**0.96**
		Mean	0.66	0.89	**0.96**
	*R* ^2^	Median	0.35	0.69	**0.93**
		Mean	0.43	0.79	**0.92**
MF	PCC	Median	0.43	0.61	**0.94**
		Mean	0.48	0.66	**0.95**
	*R* ^2^	Median	0.17	0.37	**0.87**
		Mean	0.23	0.44	**0.91**

The highest values were highlighted in boldface

All the results indicate that *PhenoNet* can calculate the similarity between phenotypes effectively and has stronger correlation with GO term-based similarity than other existing methods.

### Performance evaluation on co-expression dataset

To evaluate the performance of *PhenoNet*, we also used the expression similarity of genes associated with two phenotypes as an independent evidence to test the compared three methods. This evaluation method was borrowed from previous research [20].

We first computed the *Spearman* correlation coefficient *ρ*(*g*_*i*_,*g*_*j*_) [54] between gene *g*_*i*_ and gene *g*_*j*_. Given two phenotypes *p*_1_ and *p*_2_, let *G*_1_ and *G*_2_ be the protein sets associated with *p*_1_ and *p*_2_ respectively. The expression similarity between two phenotypes is defined as the average of |*ρ*(*g*_*i*_,*g*_*j*_)| over all protein pairs between *G*_1_ and *G*_2_ (Eq. 11).

11 $$ {{Sim}_{phe}(p1,p2)} = \frac{1}{{|G_{1}|\times|G_{2}|}} \sum_{g_{i} \in G_{1}, g_{j} \in G_{2}} |\rho(g_{i},g_{j})|  $$

**Fig. 9:**
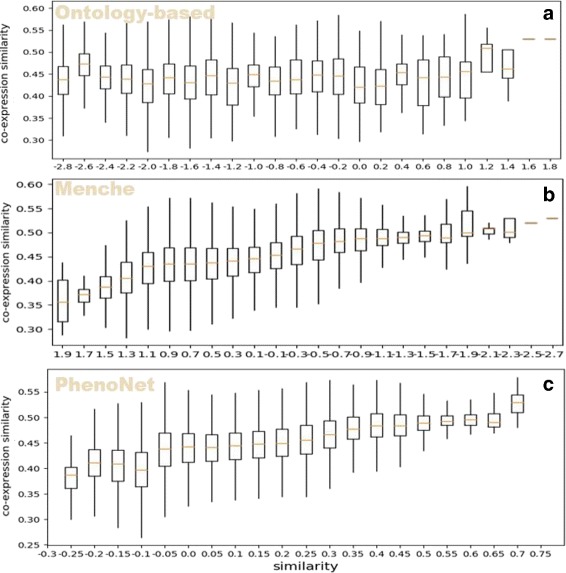
The comparison among three methods evaluated by gene co-expression analysis. The x-axis is the result of these three methods: ontology-based (**a**), Menche’s method (**b**) and *PhenoNet* (**c**). The y-axis is gene co-expression similarity

We also compared our method with ontology-based and Menche’s method. The result shows that both *PhenoNet* and Menche’s method are correlated with the expression similarity (Fig. 9). There is no correlation trend between ontology-based method and expression similarity. In general, *PhenoNet* performs better than other methods. We also calculated *Pearson* correlation coefficient (*PCC*) scores of every method on the median and mean value of scores in each bar in Fig. 9. *Pearson* correlation coefficient (*PCC*) score of *PhenoNet* on median is 0.96, while the scores of Menche’s method and ontology-based method are 0.93 and 0.42 respectively. *Pearson* correlation coefficient (*PCC*) score of *PhenoNet* on mean is 0.97, while the scores of Menche’s method and ontology-based method are 0.94 and 0.42 respectively (Table 2). In addition, we also calculated the *R*^2^ score for each method. The *R*^2^ scores of *PhenoNet* on median and mean value are 0.93 and 0.95, which are higher than the second best Menche’s method (0.86 and 0.90 respectively).

**Table 2:** The Pearson correlation coefficient and *R*^2^ score between three methods and gene co-expression similarity

Measure		Ontology-based	Menche	PhenoNet
PCC	Median	0.42	0.93	**0.96**
	Mean	0.42	0.94	**0.97**
*R* ^2^	Median	0.16	0.86	**0.93**
	Mean	0.17	0.90	**0.95**

The highest values were highlighted in boldface

These results indicate that *PhenoNet* can calculate the similarity between phenotypes effectively and has stronger correlation with expression similarity than other existing methods.

### Significance of differentiating similar and dissimilar phenotypes

**Fig. 10:**
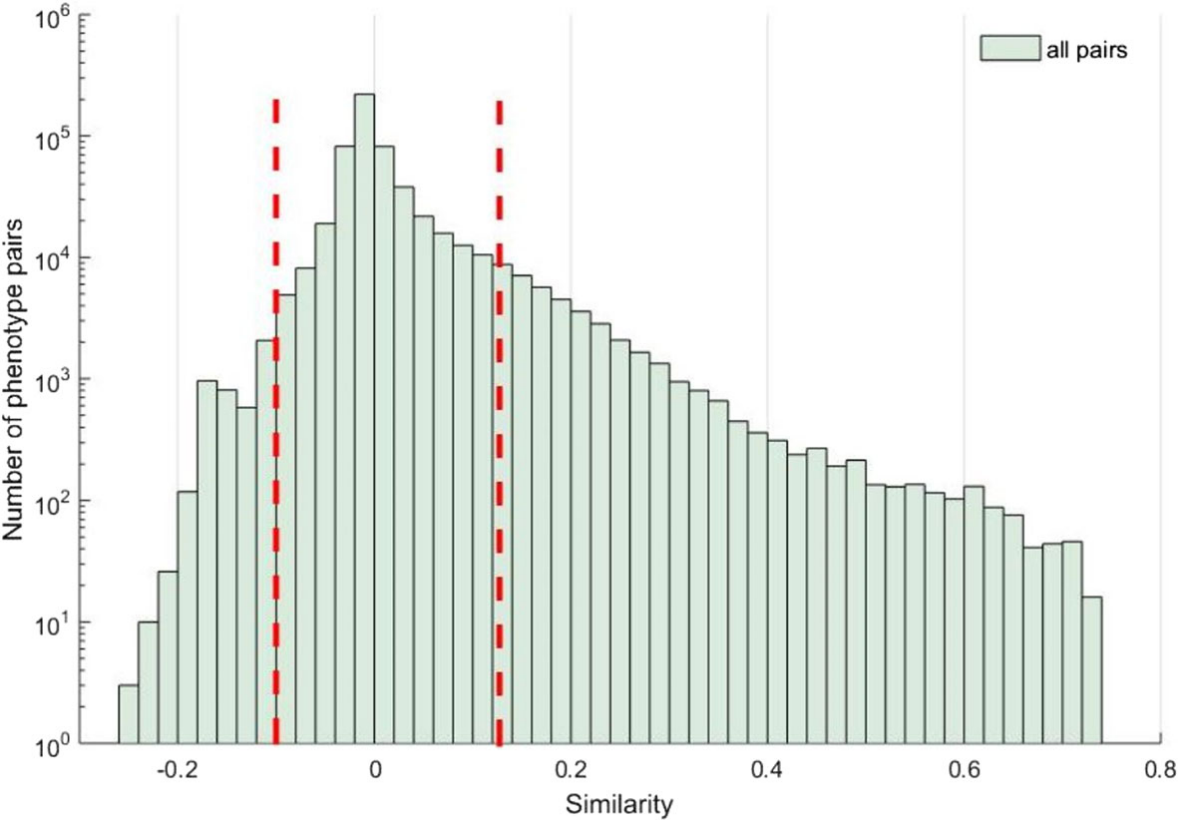
Similarity distribution of all phenotype pairs. The similarity ranges from -0.25 to 0.75, and the red lines indicate the boundary similarity scores of significantly similar or dissimilar pairs

To test whether *PhenoNet* can differentiate similar and dissimilar phenotypes efficiently, we compared the *PhenoNet* score of similar phenotype pairs and dissimilar phenotype pairs. The phenotype pairs with “high” and “low” *PhenoNet* scores are defined as similar and dissimilar set respectively. Specifically, the similar and dissimilar phenotype pairs are defined based on the following statistical model. Firstly, 100,000 pairs of phenotypes are selected randomly, saved as *P*_*ran*_. The similarities of phenotype pairs in *P*_*ran*_ are calculated to obtain the distribution of the similarities. Secondly, we computed the *p*-value of each pair. Phenotype pairs with *p*-value ≤− 0.05 and *p*-value ≥0.05 are defined as dissimilar and similar phenotype pairs respectively. Figure 10 shows the distribution of similarities. The two red lines in Fig. 10 represent the boundary of similarity and dissimilarity.

**Fig. 11:**
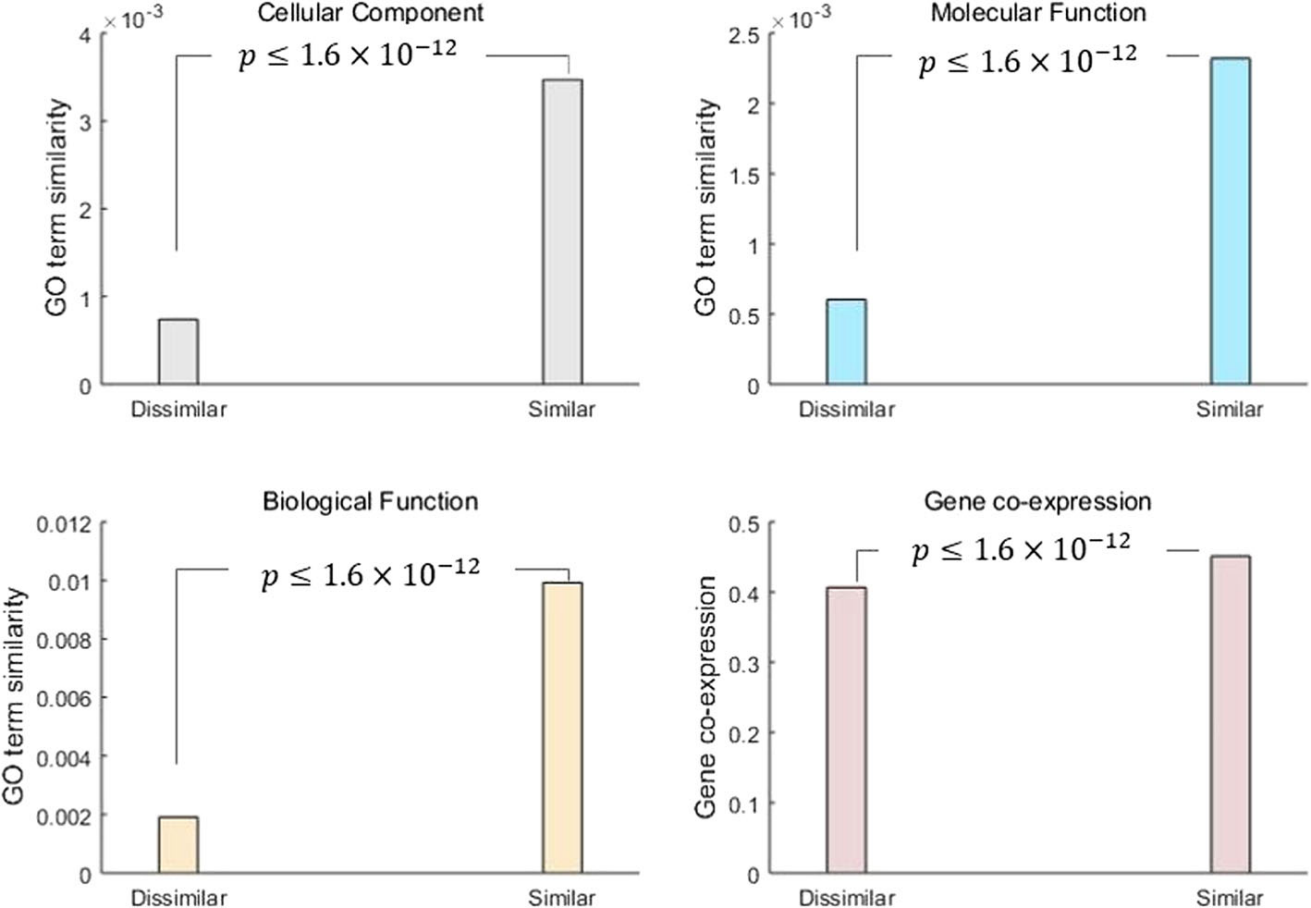
Comparison between similar and dissimilar phenotype pairs. We compare them(the x-axis) in terms of GO-based similarity and gene co-expression similarity (the y-axis). *p*-value (Mann-Whitney U test) shows the similar and dissimilar pairs of phenotypes are significantly different

After we obtain the similar and dissimilar set, we can test whether the similar and dissimilar pairs of phenotypes are significantly different based on different independent evidences. The similarities of phenotype pairs are calculated based on three GO categories (MF, CC, BP) and gene expression data respectively. Then, we test whether the similarities of similar set and dissimilar set are significantly different. The results show that the similarities of similar set and dissimilar set are significantly different (Fig. 11, Mann-Whitney U test [55], *p*-value ≤1.6×10^−12^). In Fig. 11, the bars represent the mean value of the similarities of phenotype pairs in the similar or dissimilar set. The results show that the mean values of dissimilar set are smaller than the mean values of similar set based on all four independence evidences. These results indicate that *PhenoNet* can differentiate similar and dissimilar phenotypes efficiently.

## Conclusions

Phenotype similarity calculation plays a key role in disease diagnosis. Recently, some methods have been proposed to measure the phenotype similarity. These measures can be grouped into three categories: text mining-based, ontology-based. However, the existing methods can not distinguish the similarities between terms with the same common ancestor and ignore the interactions between annotated proteins by phenotypes. In this paper, we proposed a network based method, called *PhenoNet*, to calculate the phenotype similarity. *PhenoNet* includes three steps: network modules identification, network localization, phenotype internal similarity calculation and phenotype similarity calculation. The network localization test shows that phenotypes can be represented by network modules. We compared *PhenoNet* with two existing methods: ontology-based method and Menche’s method. Furthermore, based on two independent evaluation datasets (gene ontology and gene co-expression data), evaluation test shows that *PhenoNet* performs better than existing methods. Our work opens a new window for phenotype similarity calculation, which may be potentially helpful to disease diagnosis.
